# *In utero* Exposure to Excessive Estrogen Impairs Homologous Recombination and Oogenesis via Estrogen Receptor 2 in Mice

**DOI:** 10.3389/fcell.2021.669732

**Published:** 2021-06-04

**Authors:** Xinyi Mu, Zhihan Tu, Xuemei Chen, Yi Hong, Yanqing Geng, Yan Zhang, Xingduo Ji, Taihang Liu, Yingxiong Wang, Junlin He

**Affiliations:** ^1^College of Basic Medicine, Chongqing Medical University, Chongqing, China; ^2^Joint International Research Laboratory of Reproduction and Development, Chongqing Medical University, Chongqing, China; ^3^Laboratory of Reproductive Biology, School of Public Health and Management, Chongqing Medical University, Chongqing, China

**Keywords:** estrogen, meiotic prophase I, homologous recombination, oocytes, ovarian follicle, estrogen receptor 2

## Abstract

The association between the accumulation of synthetic chemicals with estrogenic activity and risks to oogenesis has become a growing concern. This study indicates that *in utero* estrogen exposure can affect homologous recombination in early oogenesis and influence the reproductive potential and lifespan of female offspring. We conducted this study in developing mouse ovaries using two different models: oral doses administered to the mother, and fetal ovary cultures. Our analyses of meiotic fetal oocytes suggest that 17-β-estradiol induces gross aberrations in prophase I events, including delayed meiotic progression, increased unrepaired DNA damage, and altered homologous recombination levels. These effects were mainly mediated by estrogen receptor 2 (ESR2) activation. Mid-gestation exposure to estrogen also led to delayed primordial folliculogenesis after birth, impaired follicle development after prepuberty, and ultimately reduced the total litter size of the offspring. This raises the concern that maternal exposures to substances activating ESR2 may compromise the fertility of the exposed female fetus.

## Introduction

A variety of synthetic chemicals that mimic the actions of estrogen are widely used in industrial and consumer products, resulting in a nearly continuous exposure in humans. Being a fundamental organ for oogenesis and steroidogenesis, the ovary has gained unprecedented levels of attention as it is targeted by these chemicals. Such chemicals therefore pose major risks toward women’s health, as made apparent from abundant experimental models and epidemiologic studies (Patel et al., 2015). In addition, there is increasing concern regarding the effect of such exposure on early oogenesis during fetal life, as the entire ovarian reserve is established during this early stage, and disruption may lead to long-lasting consequences in the reproductive lifespan (Johansson et al., 2017).

Homologous recombination during meiotic prophase I (MPI) is a critical step of early oogenesis in the fetal stage. The chromosomes go through a unique succession of events during MPI. First, germ cells enter the leptotene stage, and the topoisomerase-like protein, Spo11, is recruited to the DNA to induce double-strand breaks (DSBs), initiating homologous recombination and homolog pairing at chromosomal ends. At the zygotene stage, aligned homologs begin connecting with each other to form ladder-like structures called the synaptonemal complex (SC), through a process termed synapsis. Then, at the pachytene stage, homologous chromosome pairs become completely synapsed and DSBs are fully repaired with a subset that has exchanged chromosomal segments and formed crossovers, which indicates completion of homologous recombination. At the diplotene stage, homologs are dissociated, apart from the notable exception at crossover sites. Evidently, homologous recombination is essential in that it generates genetic variations within a species and produces crossovers that physically hold homologous chromosomes together, ensuring proper chromosome segregation in the first division of meiosis (Reichman et al., 2017).

The accurate execution of homologous recombination is crucial for producing healthy gametes. Meiotic aberrations such as abnormal pairing, improper synapsis, and disrupted DSB repair result in meiotic arrest or cell death. In addition, they impair the ability of crossover formation, giving rise to chromosome missegregation during meiotic division, which is a leading cause of aneuploidy (Reichman et al., 2017). Unlike spermatogenesis, in which waves of spermatogonium continuously initiate meiosis after puberty, oocytes go through MPI and are then arrested at the diplotene stage, which occurs a few days before birth in mice and during the second pregenant trimester in humans. Diplotene oocytes remain dormant until stimulated by cyclic surges of gonadotropins, after entering puberty. Therefore, the seemingly irrelevant MPI events that occur during fetal development are in fact a highly vulnerable window for oogenesis. Emerging evidence indicates that exposure to chemicals such as bisphenol A (BPA) (Susiarjo et al., 2007), Diethylhexyl Phthalate (DEHP) (Liu J.C. et al., 2017), Dibutyl phthalate (DBP) (Tu et al., 2019), and Zearalenone (ZEA) (Liu K.H. et al., 2017) cause gross meiotic aberrations on MI progression and increase the likelihood of aneuploid eggs and embryos. These chemicals interfere with estrogen receptors (ESRs), and thus induce estrogenic activity, with some even up-regulating ESR expression (Liu J.C. et al., 2017). Moreover, estrogen receptor 2 (ESR2) knockout oocytes experienced multiple synapsis aberrations and increased recombination (Susiarjo et al., 2007), further suggesting a compelling role of estrogen in this process. In this regard, understanding the influence of estrogen on the early stages of oogenesis is significant in evaluating the potential risk of estrogenic chemicals.

In the present study, we hypothesize that fetal ovaries exposure to additional 17-β-estradiol (E_2_) may impair the ongoing MPI, and subsequently result in compromised female fertility. To this end, pregnant mice were administered by oral doses of E_2_, and fetal ovaries were also cultured and treated with E_2_. We report that exposure to excessive estrogen delays meiotic progression in MPI, and causes an ESR2-mediated disruption of homologous recombination in oocytes. Furthermore, exposed offspring exhibited impaired folliculogenesis and reduced fertility.

## Results

### *In utero* Exposure to Excessive Estrogen Delays Meiotic Prophase I Progression in Female Mice

**FIGURE 1:**
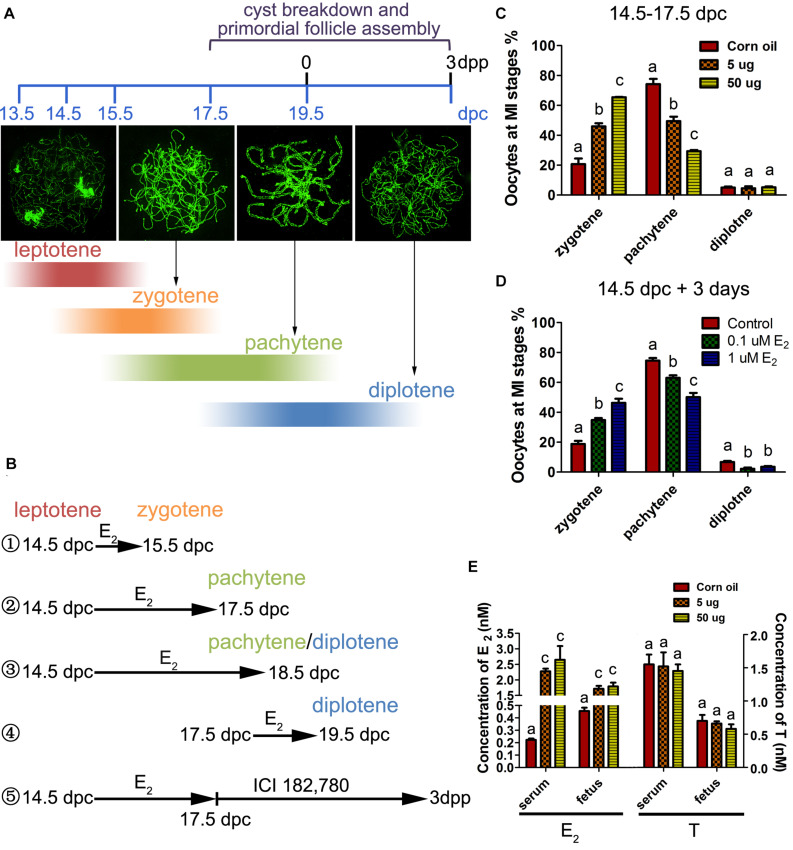
Excessive E_2_ delays meiotic prophase I progression in fetal oocytes. **(A)** The key developmental events of ovaries from 13.5 dpc to 3 dpp. Typical chromosome images of the MPI substages stained by SYCP3 were shown. The color depth of the bands indicates the proportion of oocytes at each substage occupied in the developing ovary corresponding to the timeline. **(B)** Different treatment time were adopted in sets of experiments according to the MPI progression to investigate the sensitive window of meiotic oocytes to E_2_. ① 14.5-15.5 dpc: most fetal oocytes will progress from leptotene to zytotene; ② 14.5-17.5 dpc: most fetal oocytes will progress from leptotene to pachytene; ③ 14.5-18.5 dpc: most fetal oocytes will progress from leptotene to pachytene or diplotene; ④ 17.5-19.5 dpc: most fetal oocytes will progress from pachytene to diplotene; ⑤ Fetal ovaries were treated with E_2_ from 14.5 to 17.5 dpc, and the effect of E_2_ was eliminated by ICI 182,780 from 17.5 dpc to 3dpp when primordial folliculogenesis was ongoing. **(C)** Maternal exposure of E_2_ delayed MPI progression of fetal oocytes. **(D)** E_2_ delayed MPI progression in fetal ovary culture. **(E)** The levels of E_2_ and Testosterone in the mothers’ serum and fetuses. Different letters: *P* < 0.05.

Oocytes initiate meiosis 13.5 days post coitus (dpc), and enter the leptotene stage by 14.5 dpc. By 17.5 dpc, pachytene stage cells are predominant in the ovary and homologous recombination is completed (Sun and Cohen, 2013). To assess the effect of excessive estrogen on MPI, E_2_ (5 or 50 μg/g⋅bw/day) was administered to pregnant mice of 14.5 dpc for 3 days. First, the levels of E_2_ and testosterone (T) were investigated in the mother mice’s serum and the fetuses at 17.5 dpc by ELISA. The E_2_ levels remarkably increased by the oral administration both in mothers and fetuses, while the T levels remained constant, suggesting steroidogenesis of the mothers were not affected (Figure 1E). Then, the preparations of chromosome spreads were made at 17.5 dpc (Figure 1B②). The exact stage of individual oocytes during MPI was determined according to the appearance of axial elements (Figure 1A). We analyzed the relative proportion of cells in each substage and found that there was an abundance of oocytes arrested in the zygotene stage with incomplete synapsed chromosomes, and fewer oocytes in the later meiotic stages, at both dosages of E_2_ (Figure 1C).

To determine whether the delay of MPI was a direct effect of E_2_ overexposure, 14.5 dpc fetal ovaries were cultured with 0.1 or 1 μM of E_2_ for 3 days (equal to 17.5 dpc). Consistent with maternal exposure, fetal ovary exposure to E_2_ inhibited meiotic progression (Figure 1D). When the culture was prolonged for a total of 4 days (Figure 1B③), nearly half of the zygotene oocytes still colonized the 1 μM E_2_-treated ovary (Supplementary Figure 1). In addition, in 14.5 dpc ovaries cultured for 1 day (Figure 1B①), and in 17.5 dpc ovaries cultured for 3 days (Figure 1B④), meiotic progression was unaffected by E_2_ (Supplementary Figure 1). Therefore, the delay of MPI progression by E_2_ was solely restricted to the transition from the zygotene substage to the pachytene substage. Hereafter, 5 μg/g⋅bw/day or 1 μM E_2_ dosages were used to explore further mechanisms.

### The ESR2 Is Essential for Regulating Meiotic Prophase I in Females

**FIGURE 2:**
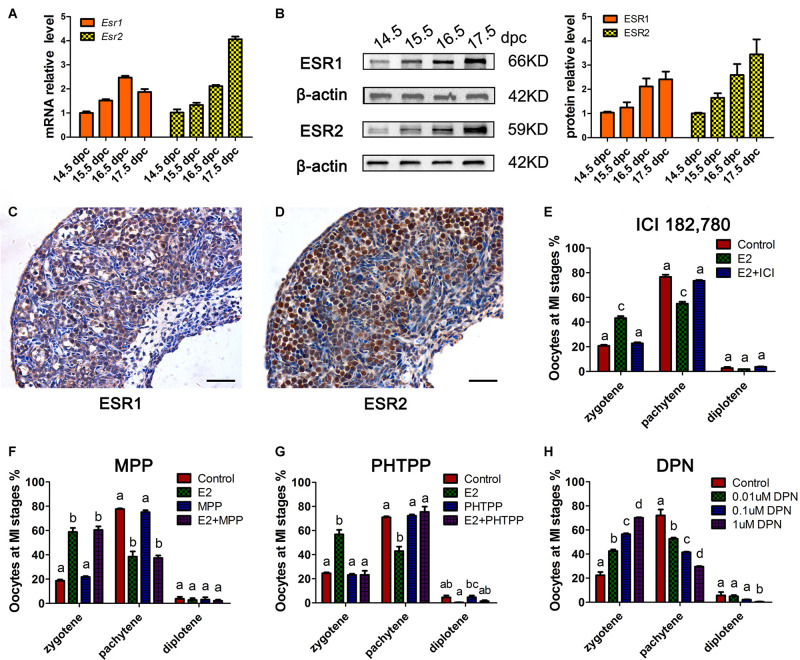
ESR2 regulates meiotic prophase I of fetal oocytes. The mRNA **(A)** and protein **(B)** expressions of ESR1 and ESR2 in the developing fetal ovary. The distribution of ESR1 **(C)** and ESR2 **(D)** in 15.5 dpc ovary by IHC. ICI 182,780 **(E)** and PHTPP **(G)** recovered the delaying effect of E_2_ on MPI. **(F)** MPP didn’t affect the delaying effect of E_2_ on MPI. **(H)** DPN delayed the MPI progression. Different letters: *P* < 0.05.

To determine whether the classic nuclear receptors of estrogen contribute to the regulation of MPI, their expression was investigated in 14.5–17.5 dpc ovaries. mRNA of *Esr1* and *Esr2* were constantly expressed and ESR1 and ESR2 protein expression increased throughout ovary development (Figures 2A,B and Supplementary Figure 2). Specifically, ESR1 was localized in the cytoplasm of oocytes, while ESR2 was detected in the nuclei of oocytes (Figures 2C,D). The distinct patterns of these two ESRs in the developing ovary may remain after birth (Chen et al., 2009).

Subsequently, ICI 182,780 (fulvestrant), a widely used inhibitor of both ESR1 and ESR2, was added to the ovary culture. We found that ICI 182,780 efficiently reversed the delaying effect of estrogen (Figure 2E). To determine the involvement of each ESR in MPI, MPP, an ESR1-selective antagonist, and PHTPP, an ESR2-selective antagonist were employed. Consistent with a previous report that BPA disrupts MPI by interfering with the actions of ESR2 (Susiarjo et al., 2007), PHTPP fully reversed the effect of E_2_ on MPI (Figure 2G), while the combined treatment with E_2_ and MPP still showed a delayed effect on meiotic progression (Figure 2F).

To further confirm the role of ESR2, DPN, a highly potent ESR2 agonist was added to the culture. To our expectation, DPN arrested oocytes in an earlier meiotic stage in a dose-dependent manner (Figure 2H). Notably, with only one-tenth that of E_2_ dosage, 0.1 μM DPN exerted a similar effect on oocyte meiosis (Supplementary Figure 2). Therefore, 0.1 μM DPN were used to explore the involvement of ESR2 hereafter. These results, utilizing specific ESR antagonists and agonist, indicated the major role of ESR2 in mediating the effect of E_2_ on MPI.

### Excessive Activation of ESR2 Disrupts Homologous Recombination

From the leptotene to the pachytene substage, the meiotic chromosomes synapse between homologs and undergo homologous recombination in an orchestrated manner. Besides, recombination is required for synapsis in mice (Bolcun-Filas et al., 2014). To test the hypothesis that excessive E_2_-induced zygotene arrest is associated with impaired homologous recombination, the repair of DSBs and crossover formation were examined by superresolution structured illumination microscope (SIM) in pachytene oocytes.

**FIGURE 3:**
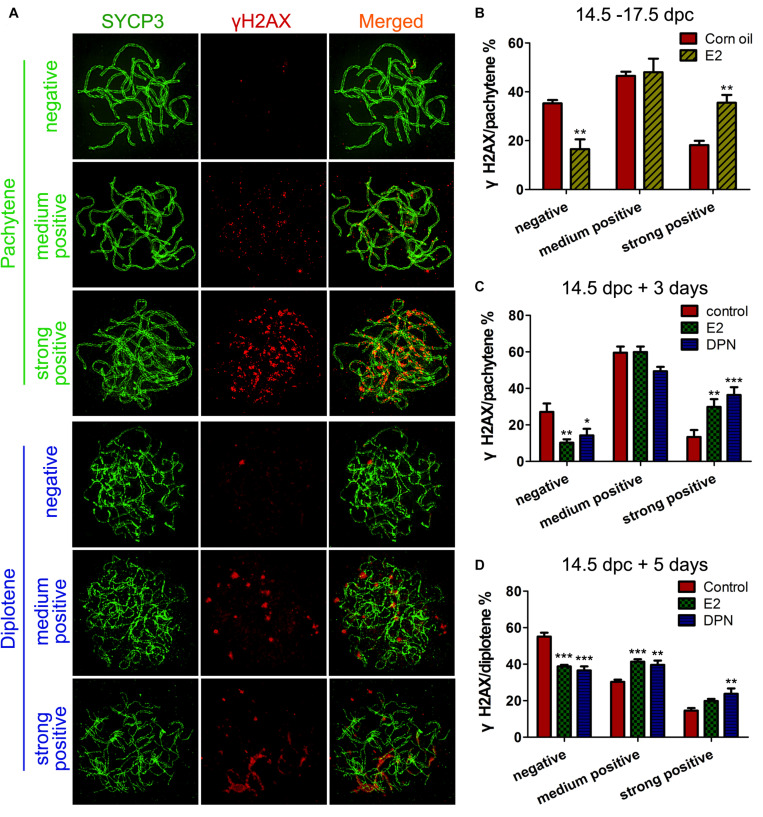
E_2_ Disrupts γH2AX pattern in fetal oocytes. **(A)** Three classes of γH2AX pattern in pachytene and diptotene oocytes. **(B)** The effect of *In utero* exposure of E_2_ on γH2AX patterns of pachytene oocytes. **(C)** The effect of E_2_ and DPN on γH2AX patterns of pachytene oocytes in ovary cultures. **(D)** The effect of E_2_ and DPN on γH2AX patterns of diplotene oocytes in ovary cultures. **P < 0.01. ***P < 0.001.

Firstly, we observed the staining pattern and intensity of γH2AX, a DSB marker, in pachytene oocytes, and defined them as three main classes: negative, medium positive (a few small foci), and strong positive (numerous foci and fragment-like foci) (Figure 3A). After 3 days of *in utero* exposure of E_2_, the percentage of γH2AX-negative pachytene oocytes was significantly decreased, and strong-positive pachytene oocytes was remarkably increased in the fetal ovary (Figure 3B). The evaluation of γH2AX staining was further verified in cultured ovaries, and a very similar result was observed: 3 days of culture with E_2_ or DPN remarkably reduced γH2AX-negative pachytene oocytes, and increased the proportion of strong-positive cells (Figure 3C). This difference became further evident in analysis restricted to diplotene oocytes when 14.5 dpc ovaries were cultured for 5 days. In addition to the reduction of γH2AX-negative oocytes and the increase of strongly positive oocytes, the medium-positive cells for γH2AX were also markedly elevated (Figure 3D). The altered presence of γH2AX in pachytene and diplotene oocytes reflected defective DSB repair.

**FIGURE 4:**
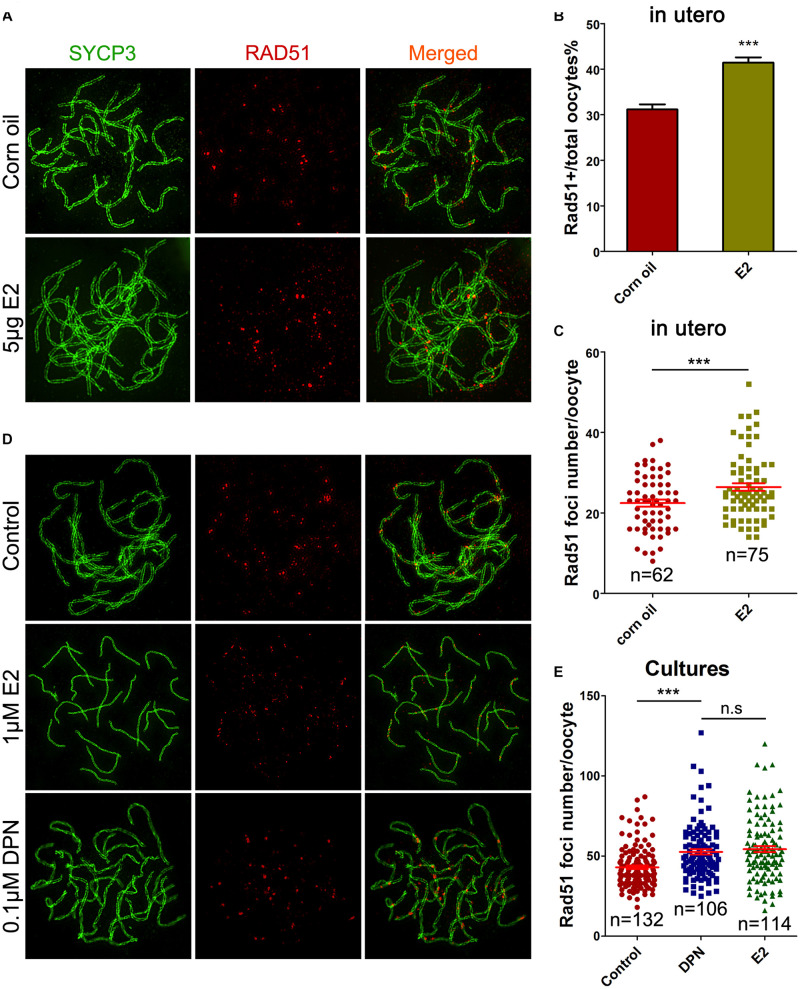
E_2_ increases RAD51 foci in fetal oocytes. **(A–C)** Pachytene oocytes received *in utero* exposure. **(A)** RAD51 foci were indicated by co-staining of SYCP3 (Green) and RAD51 (red). **(B)** E_2_ increased the percentage of RAD51-positive oocytes. **(C)** E_2_ increased RAD51 foci number. **(D,E)**: Pachytene oocytes from ovary culture. **(D)** Co-staining of SYCP3 (Green) and RAD51 (red). **(E)** E_2_ and DPN increased RAD51 foci number. ***P < 0.001. n.s: not significant.

Evidence of excessive E_2_ causing unrepaired DNA damage in late MPI oocytes was obtained by staining for Rad51, which binds to DSBs and plays critical roles in catalyzing homologous pairing, DNA strand exchange and DSB repair (Reichman et al., 2017). The pachytene oocyte with increased Rad51 foci on the SCs can be considered deficient in DSB repair. After maternal oral administration of E_2_, with a significant increase in the proportion of Rad51-positive pachytene oocytes (Figure 4B), the number of Rad51 foci per pachytene oocyte was remarkably increased (Figures 4A,C). Moreover, consistent findings were observed in cultured ovaries where administration of E_2_ or DPN significantly increased Rad51 counts in pachytene cells (Figures 4D,E). The abnormal increases in γH2AX and Rad51 staining both signified a failure to resolve DSBs in late MPI.

**FIGURE 5:**
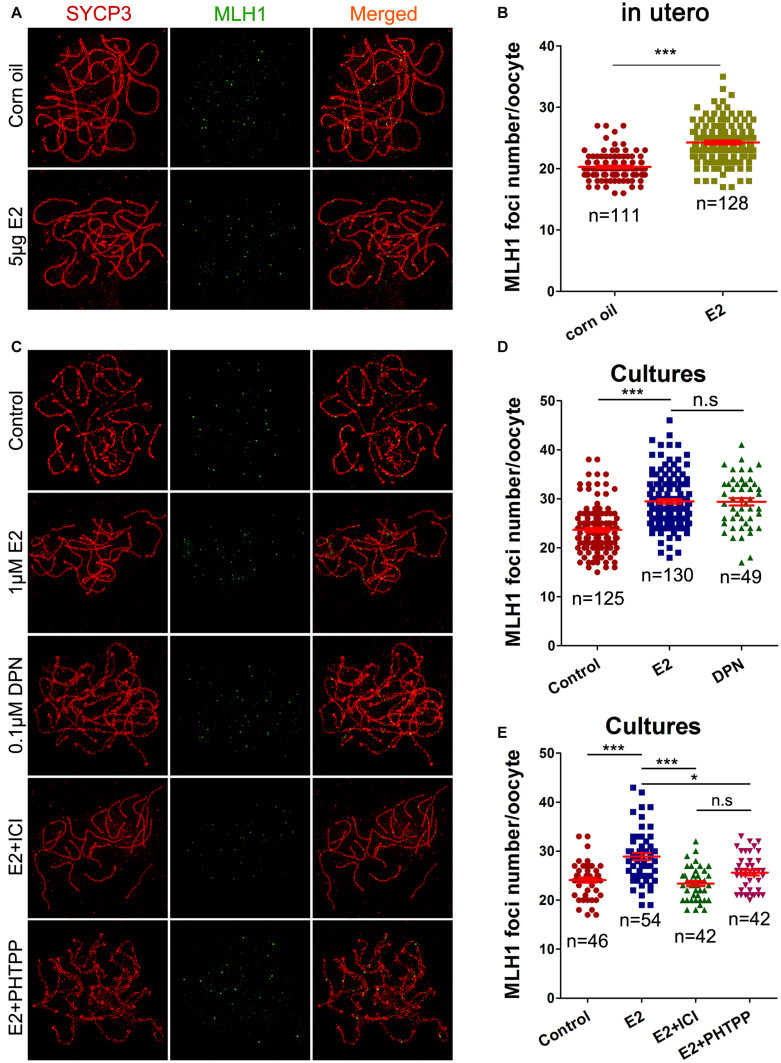
E_2_ increases MLH1 foci in fetal oocytes mainly by ESR2. **(A,B)**: pachytene oocytes received *in utero* exposure. **(A)** MLH1 foci were indicated by co-staining of SYCP3 (red) and MLH1 (green). **(B)** E_2_ increased MLH1 foci number. **(C–E)**: pachytene oocytes from ovary culture. **(C)** Co-staining of SYCP3 (red) and MLH1 (green). **(D)** E_2_ and DPN increased MLH1 foci number. **(E)** ICI 182,780 and PHTPP reversed the increased MLH1 counts. *P < 0.05. ***P < 0.001. n.s: not significant.

Pachytene oocytes exposed to excessive E_2_ also displayed striking aberrations in the formation of crossovers, as assessed by the number of MLH1 foci along the SCs. As a component of the post-replicative DNA mismatch repair system, most meiotic crossovers arise by the action of the MLH1, and the counts of MLH1 foci are frequently used to assess homologous recombination levels (Reichman et al., 2017). Following oral administration, MLH1 counts per pachytene cell were significantly higher in the E_2_ group than in the control (Figures 5A,B). Consistently, a significant increase in mean MLH1 values was also observed in ovary cultures with E_2_ or DPN than in the control (Figures 5C,D).

As DPN showed similar effects to E_2_ at one-tenth of the concentration in all the above assessments of homologous recombination, the involvement of ESR2 was further investigated in ovary cultures. Ovaries of 14.5 dpc were administrated to E_2_, with a pre-treatment with ICI 182,780 or PHTPP. As expected, both ICI 182,780 and PHTPP significantly reversed the increased MLH1 counts caused by estrogen, producing no significant differences from the control (Figures 5C,E). It further suggested the dominant role of ESR2 in mediating the effect of E_2_ on homologous recombination.

### Excessive E_2_ Represses the Expression of Various Meiosis-Related Genes in Fetal Ovaries

To gain a better understanding of the potential molecular functions of E_2_ in oogenesis, we explored multiple meiotic regulators and effectors during fetal ovarian development.

**FIGURE 6:**
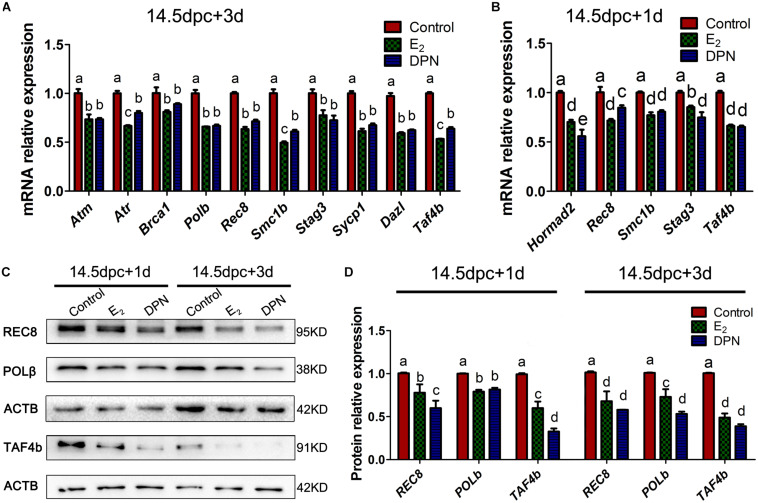
Activation of ESR2 Disrupts expressions of key meiotic genes. Ovaries of 14.5 dpc were cultured. E2 and DPN decreased the mRNA expressions of key meiotic genes in cultures for 3 days **(A)** or 1 day **(B)**. E2 and DPN decreased the protein expressions of key meiotic genes in cultures for 1 day or 3 days **(C,D)**. Different letters: *P* < 0.05.

Quantitative PCR revealed that after 3 days of culturing 14.5 dpc ovaries, the E_2_ and DPN treatment downregulated the mRNA expression of *Atm*, *Atr*, *Brca1*, *Polb*, *Rec8*, *Smc1b*, *Stag3*, *Sycp1*, *Dazl*, and *Taf4b* (Figure 6A), while expressions of *Chek2*, *Hormad1/2*, *Dmc1*, *Sun1*, *Trip13*, *Rad21*, *Rad21l*, and *Smc3* showed no significant difference (Supplementary Figure 3). As the reduction of gene expression may be due to the varied population of meiotic substages, and the fact that the expression of several essential genes are restricted to early MPI and are downregulated by 16.5 dpc (Soh et al., 2015), we further examined the mRNA expression in 14.5 dpc ovaries cultured for 1 day. This ensured that the overall meiotic progression was not affected (Supplementary Figure 1). Similarly, it was found that E_2_ and DPN significantly reduced the mRNA expression of *Rec8*, *Smc1b*, *Stag3, Taf4b*, but also *Hormad2* (Figure 6B), while the expression of other genes remained constant. The reduced expression of POLb, REC8, and TAF4b was further confirmed by western blotting (Figures 6C,D). The decrease in meiotic gene expression further indicates that meiotic progression defects are caused by estrogen.

### Excessive Exposure to E_2_ During Meiotic Prophase I Impairs Germline Cyst Breakdown and Primordial Follicle Formation

**FIGURE 7:**
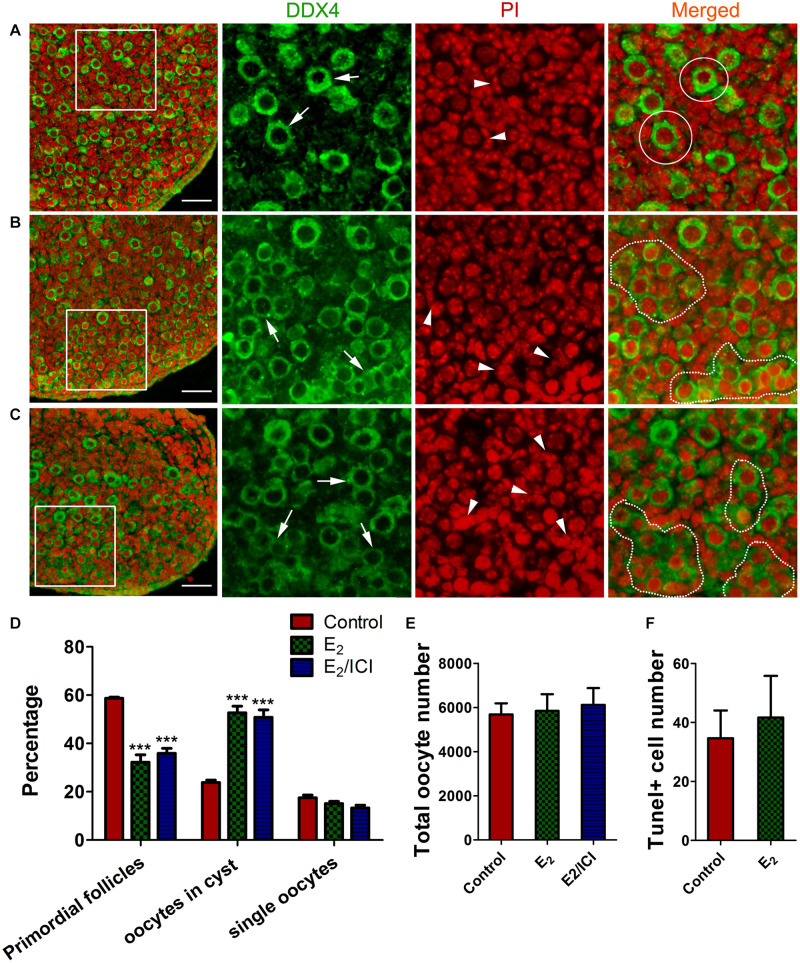
E_2_-delayed meiotic prophase I impairs primordial follicle assembly. Sections from control group **(A)**, E_2_ group **(B)** and E_2_ + ICI 182,780 group **(C)** were stained with DDX4 (green) to visualize oocytes and PI (red) to visualize nucleus. Framed area was enlarged. Arrow: oocyte, arrowhead: pregranulosa cell, White circle: primordial follicle, dotted line: germ-cell cyst. Scale bar, 40 μm. Counting analysis for primordial follicles assembly **(D)**, total oocytes **(E)** and TUNEL-positive oocytes **(F)**. ***P < 0.001.

We investigated potential effects that the meiotic-phase exposure of high E_2_ might have on controlling early folliculogenesis. Ovaries of 14.5 dpc were cultured with E_2_ for 3 days, and for a further 5 days without E_2_, aging the ovaries to 3 dpp. Then, cyst breakdown, primordial follicle formation, and oocyte counts were analyzed. Slides were stained with PI for nucleus and DDX4 for oocytes, so that primordial follicle surrounded by a single layer of pregranulosa cells (Figure 7A) and germ cell cysts with connected cytoplasm can be easily recognized (Figures 7B,C).

E_2_-treatment remakably delayed primordial follicle formation, with half of the oocytes were still in the germ cell cysts (Figures 7B,D). As postnatal exposure to E_2_ delays primordial follicle formation (Chen et al., 2007), ICI 182,780 was included in the final 5 day culture in order to eliminate the effect of E_2_ during the time (Figure 1B⑤). Primordial follicle formation was not recovered, and the proportion of cysts oocytes was similar to that of the E_2_-treated ovaries (Figures 7C,D). This indicated that excessive E_2_ during MPI plays a role in controlling the subsequent assembly of primordial follicles.

Unexpectedly, the total oocyte number in all groups was approximately the same (Figure 7E), indicating that oocyte survival was not affected by the E_2_-induced meiotic abnormality. This observation was further confirmed by TUNEL assay that only a few oocytes underwent apoptosis and there were no significant differences in positive-staining oocyte counts (Figure 7F and Supplementary Figure 4). This implied the possibility of insufficient activation of the meiotic checkpoint, which raises the risk for the ovulation of oocytes with genetic defects in postpubescent female offspring.

### *In utero* Exposure to Excessive E_2_ Damages Long-Term Female Fertility

**FIGURE 8:**
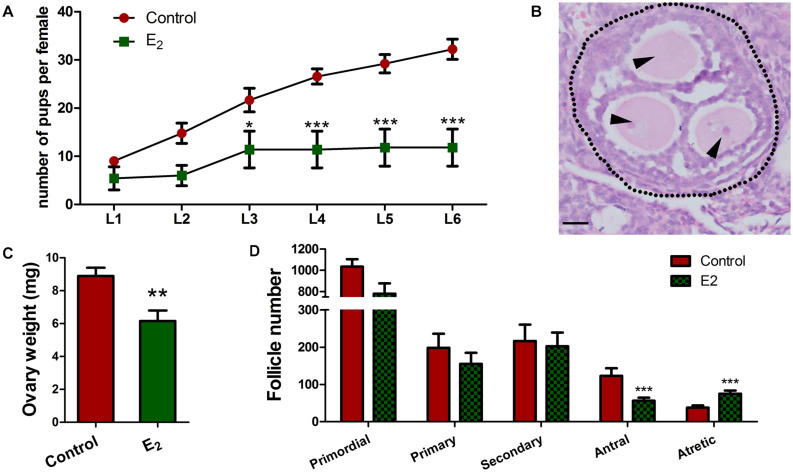
*In utero* exposure of excessive E_2_ compromises female fertility. **(A)** Female F1 with *in utero* exposure of E_2_ gave birth to fewer pups. **(B–D)** Prepubertal F1 females were injected with PMSG. **(B)** An MOF from a mouse *in utero*-exposed to E_2_. Dotted line: follicle wall, arrowhead: oocyte. Bar: 20 μm. The effect of *in utero* exposure of E_2_ on ovary weight **(C)**. Counting analysis for follicle development **(D)**. Bar: 50 μm. *p < 0.05, **p < 0.01, ***p < 0.001.

Since the retention of meiotic aberrations in early oogenesis raises the production of aneuploid oocytes and genetic defects, the fertility of female offspring was evaluated. In each group, three pregnant mice (F0) fed with corn oil or E_2_ were allowed to go to full term. Female offspring (F1) were raised for 6 weeks, and then, mated with normal adult males over a 6-month period. From the third litter onward, F1 females with *in utero* exposure to E_2_ had significantly fewer pups, and some of them became completely sterile after that (Figure 8A and Supplementary Table 1). The total offspring numbers of F1 was further analyzed by a mixed model with the F0 mothers as a covariate. Consistently, it showed that the offspring of E_2_-exposed mice were significantly decreased (Supplementary Table 2). This phenotype indicates that *in utero* exposure to excessive E_2_ results in the loss of female fertility in early adulthood. Nevertheless, the male:female ratio and the birth weight of F2 pups were not altered (Supplementary Figure 5).

To further explore the possibility that reduced fertility is associated with follicle development, prepubertal F1 females were injected with PMSG to stimulate follicle development. Though there were no significant differences were observed in the total follicle numbers, E_2_ exposure significantly reduced the antral follicles and remarkably elevated the atretic follicles numbers (Figure 8D and Supplementary Table 3), and the weight of the ovaries significantly decreased in the E_2_ group compared to the control group (Figure 8C). Meanwhile, multi-oocyte follicles (MOFs), which contain more than one oocyte within a follicle, were found in five of six mice in the E_2_-exposed group, while only two of the five mice had MOFs in the control group (Figure 8B and Supplementary Table 3). The increased prevalence of MOFs further confirmed defects in the breakdown of germ cell cysts.

**FIGURE 9:**
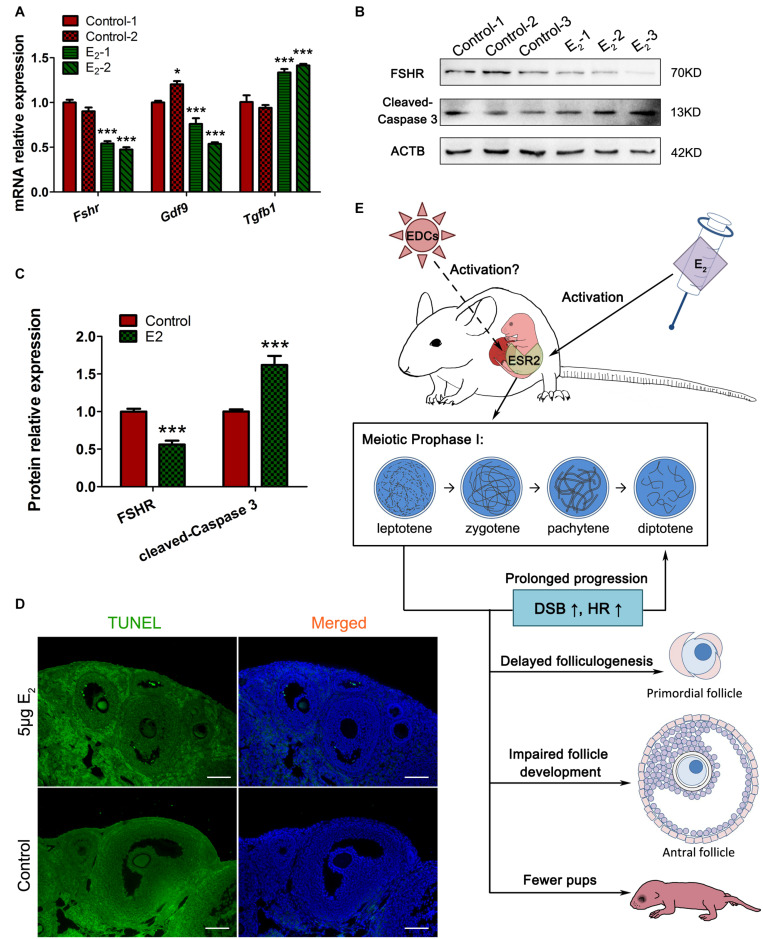
*In utero* exposure of excessive E_2_ damages antral follicle development. **(A)** The mRNA expression of key genes in antral folliculogenesis. **(B,C)** The protein expression of FSHR and cleaved caspase-3. **(D)** TUNEL assay in antral follicles. **(E)** Summary effects of *in utero* exposure of excessive E_2_ on female offspring. Bar: 50 μm. *p < 0.05, **p < 0.01, ***p < 0.001.

Since antral follicle population was reduced, whose development is gonadotropin-dependent, we further explored whether the FSH signaling was retarded. The involvement of factors such as FSHR and GDF9, which are critically important during antral folliculogenesis, and serve as targets or mediators of FSH signaling (Edson et al., 2009), and TGFβ1, which inhibits follicle development in the presence of FSH (Rosairo et al., 2008), were explored in prepubertal ovaries. The decreased levels of *Fshr* and *Gdf9* and the increased level of *Tgf*β*1* suggest defects in the gonadotropin response in *in utero*-exposed mice (Figure 9A). Furthermore, the TUNEL assay showed that granulosa cells in the antral follicles underwent apoptosis in the exposed group (Figure 9D). These observations were further confirmed by the detection of FSHR and cleaved-caspase 3 protein levels (Figures 9B,C). These factors combined may contribute to the increased number of atretic follicles. The reduced overall fertility and impaired follicle development demonstrated that *in utero* exposure to excessive E_2_ has adverse long-term effects on female reproduction of the offspring.

## Discussion

The regulated program of MPI is a prerequisite for the production of gametes, while chemicals with estrogen-like activity are potentially hazardous to the successful oogenesis progression. Here, we reported an adverse effect of estrogen on early oogenesis by disturbing synapses and recombination between homologs via ESR2. Meiotic aberrations may also disturb the establishment of the primordial follicle reserve, impair gonadotropin-responsiveness of follicles, and compromise female fertility (Figure 9E).

Despite the essential role of estrogen in the regulation of oocyte growth and maturation, knowledge of its involvement in homologous recombination was previously very limited. Nevertheless, it has now been shown that chemicals with estrogen-like activity disturb MPI progression. *In utero* exposure to BPA, a spreading additive used in the production of epoxy resins and polycarbonate plastics, induced defects in synapsis and homologous recombination in fetal mouse oocytes, resulting in an increase in aneuploid eggs and embryos in offspring (Susiarjo et al., 2007). Moreover, the meiotic aberrations caused by BPA were also observed in rhesus monkeys (Hunt et al., 2012). DEHP, an extensively used plasticizer, inhibits MPI progression in the fetal ovary with increased DNA damage, apoptosis of oocytes, and expression of ERs (Liu J.C. et al., 2017). We also reported that DBP, another phthalate plasticizer, revealed similar effects to DEHP on MPI and further increased recombination levels in fetal oocytes (Tu et al., 2019). ZEA, a mycotoxin produced by fungus *Fusarium graminearum*, delays oocytes from entering the diplotene stage, activates the DNA damage repair system, and increases DSBs in the later MPI cells (Liu K.H. et al., 2017). Additionally, estrogenic chemicals also impair meiotic progression and homologous recombination during spermatogenesis in developing and adult testes (Liu et al., 2013; Vrooman et al., 2015). These findings are in agreement with our present study showing the involvement of excessive estrogen stimulation in the complex and essential process of homologous recombination.

Since all of these chemicals interfere with estrogen receptors, it strongly implies that estrogen receptors play a role in regulating homologous recombination. ESR2KO female mice presented similar meiotic defects associated with BPA exposure, whereas ESR1KO mice exhibited a phenotype parallel to wild-type (Susiarjo et al., 2007). Meanwhile, BPA exerted no additional effects in ESR2KO oocytes. This suggested an important role of ESR2 in meiotic recombination and that BPA may act as an estrogen antagonist. However, in the present study, activation of ESR2 played a dominant role in mediating an adverse effect in MPI, while oocytes in which ESR2 signaling was blocked showed no detectable meiotic defects. The seemingly divergent role of ESR2 in the regulation of homologous recombination is possibly due to the differences in overall loss of function in ESR2KO mice, and the partial but specific inhibition of function by antagonists. Despite sexual dimorphisms, our observations were also present in spermatogenesis. In the adult testicles, E_2_ and BPA exert very similar effects on meiotic progression and meiotic checkpoint activation. Consistent with the present study, ICI 182,780 reversed these effects, however, spermatogenesis continued normally when added alone (Liu et al., 2013). On all accounts, participation of ESR2 in the regulation of MPI is prominent and intriguing, although it is still premature to hypothesize how ESR2 functions in the events examined in this study, especially when the canonical role of ESR2 as a transcription factor was not investigated. The identification of ESR2 signaling pathways will provide further insight into the regulation of MPI.

Although the involvement of ESR1 in MPI has not yet been distinguished, ESR1 signaling directly interacts with the DNA damage response and DNA repair machinery in homologous recombination of hormone-dependent breast cancer (Caldon, 2014). In this scenario, active ESR1 signaling negatively regulates key effectors in the DNA damage response, including ATM, ATR, CHK1, and BRCA1, and alters DNA damage processing in order to suppress effective DNA repair without activating cell cycle checkpoints in favor of proliferation. Although the role of ESR2 is not as well understood, it is commonly recognized that ESR2 has antagonistic effects on ESR1 in many contexts, such as in DNA damage response and DNA repair during carcinogenesis (Lewandowski et al., 2005; Thomas et al., 2011). A body of emerging evidence has revealed that meiotic chromosome regulator genes, including REC8, SMC1β, RAD21L1, TEX19, HORMAD1, and SYCP3, are inappropriately activated to modulate chromosome maintenance and segregation in tumor development, maintenance, and spread (McFarlane and Wakeman, 2017). It is a thought-provoking speculation whether the abnormal expression of meiotic key factors is due to the dysregulation of ESR signaling in tumorigenesis.

Since follicle fate is dominantly dependent on the oocyte (Eppig et al., 2002), it is very likely that the aberrant follicular development, as disrupted gonadotropin responsiveness, increased apoptotic granulosa cells and atretic follicles we observed in exposed prepubertal ovaries, which may all contribute to the reduced overall fertility, is caused by defects of the oocytes. In this regard, the implications of our findings are much broader. Besides the reduction in the genetic quality of oocytes, the *in utero* exposure to chemicals that stimulates ESR2 may adversely influence the entire female reproductive lifespan.

Altogether, our study showed that *in utero* exposure to excessive E_2_ caused aberrations in DSB repair and homologous recombination, resulting in impaired fertility during the reproductive lifespan of the offspring. This conclusion is consistent with the possibility that estrogenic chemicals influence early oogenesis, which can evolve into long-lasting consequences manifesting later after puberty, or even through future generations. These findings provoke further exploration of the effects of exposure to endocrine disruptors during pregnancy.

## Materials and Methods

### Ethics Statement

All experiments were approved by the Chongqing Medical University Animal Care and Use Committee (License no. 20180228), and were performed in accordance with institutional and national guidelines and regulations that all efforts were made to minimize suffering.

### Animals

The CD1 mice were purchased from Vital River Laboratory Animal Technology Co. LTD (Beijing, China) and housed in Chongqing Medical University Animal Care Facility. Seventy 6-week-old female mice were mated with fertile adult males, and the morning with the presence of the vaginal plug was designated as 0.5 dpc. Birth usually occurred on 19.5 dpc and was designated as 0 dpp.

### E_2_ Oral Administration and Stimulation of Follicular Development

It was reported that neonatal pups subcutaneously injected with E_2_ at a dose of 5 μg/g⋅body weight/day inhibits primordial folliculogenesis (Chen et al., 2007). Considering the absorption efficiency and the transportation across the placenta, E_2_ at single doses of 5 or 50 μg/g⋅body weight/day were administered to the pregnant mice in the mornings of the 14.5, 15.5, and 16.5 dpc by gastric infusion. The control group was given an equal volume of corn oil. Twelve pregnant mice in the control and 5 μg E_2_ groups were orally administrated. Then, the female fetuses were pooled together, and fetal ovaries were collected to analyze the meiotic progression, the foci of γH2AX, RAD51, and MLH1. Three pregnant mice in each group were allowed to go to full term to investigate the long-term effect of E_2_. Six mice were under treatment of 50 μg E_2_.

Female pups of three oral-administrated pregnant mice from each group were kept to 3–4 weeks old (prepuberty, body weight 12–14 g), then stimulation of follicular growth was performed by intraperitoneal injection of 5 IU PMSG (Solarbio science and technology Co., Beijing, China). Ovaries were collected 48 h later. One of the pairs of ovaries from 5 female pups in the control group and 6 female pups in the E2 group were analyzed for morphology and the other ones were used for mRNA and protein expression.

### Fetal Ovary Organ Culture and Chemicals

Fetal ovaries from 40 pregnant mice were separated on the desired dpc, and then cultured in DMEM/F12 serum-free media (Gibco BRL, Beijing, China) in a humidified incubator.

E_2_ (Munich, Germany), ICI 182,780, MPP dihydrochloride, PHTPP and DPN (all purchased from Tocris Cookson Inc., Toronto, Canada) were firstly dissolved in DMSO. Then stock solutions were added to the media before use as desired final concentration. DMSO was added in the same concentration (≤0.1%) as vehicle controls. The final concentrations of the chemicals were determined by a previous study (Chen et al., 2009) and our preliminary study. E_2_ (0.1 and 1 μM) and DPN (0.01, 0.1, and 1 μM) were added at the start of organ culture except for antagonist studies using ICI 182,780, MPP, and PHTPP where the antagonist was added at the concentration of 1 μM first and 1 μM E2 was added 2 h later.

### Spreading of Meiotic Chromosomes

The spreadings of meiotic chromosomes were obtained as reported previously (Tu et al., 2019). Briefly, the fetal ovaries were dispersed by trypsin and hypotonic treated, then cells were transferred onto slides and fixed with 1% paraformaldehyde (PFA). Then the slides were incubated with primary antibodies (the details were listed in Supplementary Table 5). After incubation with the secondary antibodies, slides were observed by the fluorescence microscope (BX43, Olympus, Japan), or SIM (N-sim, NIKON, Japan). In meiotic analysis, over 200 oocytes were observed on each slide, and similar observations were obtained for at least three independent experiments. The images were processed by NIS-elements BR 5.01 (NIKON, Japan).

### Histology and Morphological Evaluation

Collected ovaries were fixed in 4% PFA, transferred to 70% ethanol, and embedded in paraffin. Serial sections stained with hematoxylin-eosin were ordered sequentially on the slides. The slides were observed by the microscope (BX43, Olympus, Japan) and analyzed by BX2-BSW 03.04a (Olympus, Japan).

For each prepuberty ovary, follicles were classified as primordial, primary, secondary and antral (Supplementary Figure 5). The follicles were counted in every fifth section through the entire ovary, and only healthy, non-atretic follicles with visible oocyte nuclei were scored. The presence of MOFs and atretic follicles was also recorded.

For cultured fetal ovaries, the primordial folliculogenesis was analyzed every fifth section across the entire ovary from six ovaries for each group, and the cumulative germ cell counts were multiplied by five, because four-fifths of the ovary was not analyzed.

### Immunohistochemistry and Immunofluorescence

For immunohistochemistry, after being rehydrated and antigen retrieval, the sections were incubated with the primary antibodies (the details were listed in Supplementary Table 5). The slides were then incubated with a biotinylated secondary antibody and detected by diaminobenzidine (Zhongshanjinqiao, Beijing, China). The sections were counterstained with hematoxylin.

For immunofluorescence, after incubation with the primary antibody, the slides were then incubated with FITC-conjugated secondary antibodies and stained by PI to visualize nucleus.

### Breeding Assay

By the age of 6-week-old, five female offspring (F1) originated from three different mothers received oral dose were mated with male CD1 mouse, and each provided more than 6 consecutive litters (F2). Nine Female offsprings from three mothers received corn oil were used as controls. The litter size, the birth weight and the sex ratio of the F2 were recorded.

### RNA Extraction and Quantitative RT-PCR

Total RNA was extracted using TRI reagent (Sigma-Aldrich, St. Louise, United States) according to the manufacturer’s protocol. First-strand cDNA was created by reverse transcription (PrimeScript RT Master Mix, TaKaRa, Japan). Real-time qRT-PCR was carried out by the comparative cycle threshold method using BIO-RAD system (CFX Connect, Bio-Rad, CA, United States). The mRNA levels were normalized against *Actb*. The primers are provided in the Supplementary Table 4.

### Western Blotting

Total protein was extracted and separated on 6, 10, or 12% SDS–PAGE according to the target protein MW and transferred to PVDF membranes (Bio-Rad, CA, United States). Membranes were incubated with the primary antibodies (the details were listed in Supplementary Table 5) and visualized using the chemiluminescent HRP substrate (ABclonal, Wuhan, China). ACTB was used as an internal control. Relative intensities were quantified using Quantity One 4.5 (Bio-Rad, CA, United States).

### TUNEL Assay

Ovaries were fixed and sectioned serially, and TUNEL assay was carried out following the manufacturer’s instructions (*In Situ* Cell Death Detection Kit, Roche, Mannheim, Germany). For cultured ovaries, the signal is visualized by peroxidase substrate, the total number of apoptotic oocytes in every sections of the whole ovary was counted.

### Hormone Measurement

After the oral administration, three 17.5 dpc pregnant mice in each group were anesthetized and exsanguinated through the orbital sinus. The blood was collected in sterile tubes, naturally coagulated at 4°C over night and centrifuged (3,000 g, 20 min), then the serum were obtained. Two 17.5 dpc female fetuses from each mothers were seperated, weighed, and adequately homogenized, and the supernatant were carefully collected by centrifugation (3,000 g, 20 min). E_2_ and T levels in serum and fetuses were measured by ELISA according to the manufacturer’s instructions (ELISA kit for mouse E_2_, ELISA kit for mouse T, MEIMIAN, Yancheng, China). The hormones’ concentrations were determined by reading absorbance at 450 nm with the microplate reader (Varioskan LUX, Thermo Fisher scientific, Waltham, United States).

### Statistical Analysis

For each set of experiments, independent trials were repeated at least three times with similar results; data were shown for the repeats and represented as mean ± SEM. For analysis of γH2AX patterns, RAD51, and MLH1 foci numbers, the data were collected from oocytes of 3 independent repeated experiments. Differences among groups were statistically tested by LSD *t*-test or one-way ANOVA analysis using GraphPad Prism 5 software. The mixed model method was carried out by SPSS 16.0. Comparisons were considered significant at *P* < 0.05 and highly significant at *P* < 0.01 and *P* < 0.001.

## Data Availability Statement

The raw data supporting the conclusions of this article will be made available by the authors, without undue reservation.

## Ethics Statement

The animal study was reviewed and approved by the Chongqing Medical University Animal Care and Use Committee (Lisence No.: 20180228).

## Author Contributions

MX, TZ, ZY, CX, and LT: methodology; MX, TZ, and GY: formal analysis and investigation; MX, TZ, HY, JX, ZY, and CX: material preparation. MX, HJ, and WY: funding acquisition. MX and HJ: supervision. The first draft of the manuscript was written by MX. All authors commented on previous versions of the manuscript, read and approved the final manuscript, and contributed to the study conception and design.

## Conflict of Interest

The authors declare that the research was conducted in the absence of any commercial or financial relationships that could be construed as a potential conflict of interest.
