# Supplementary material for: In utero Exposure to Excessive Estrogen Impairs Homologous Recombination and Oogenesis via Estrogen Receptor 2 in Mice
Source: Front Cell Dev Biol. 2021 Jun 4;9:669732. doi: 10.3389/fcell.2021.669732 (PMC8212019; doi:10.3389/fcell.2021.669732)
Supplement: Supplementary file 1 [file Data_Sheet_1.docx]

Supplementary Material

**1 Supplementary Figures**

**
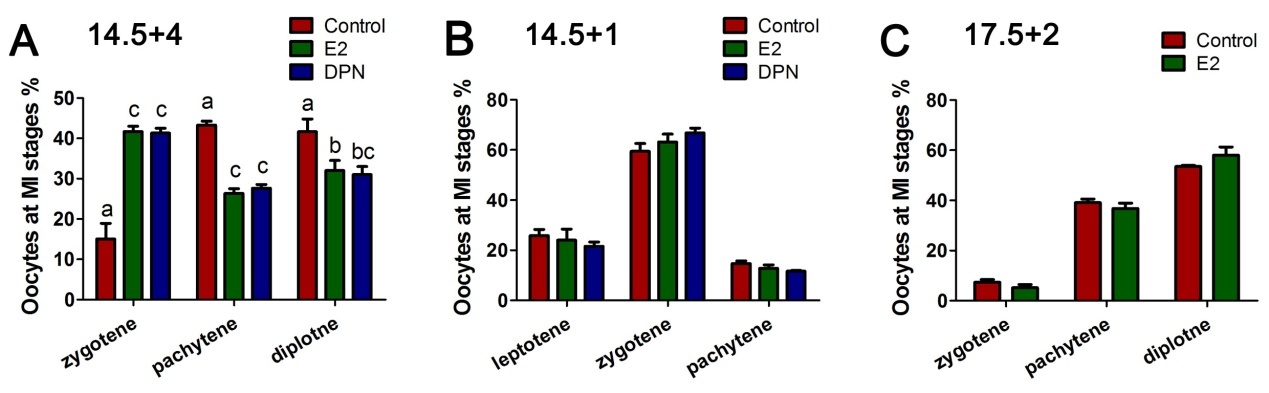
Supplementary figure 1. The different effects of E_2_ on prophase I of oocytes depend on the treatment period.** A. Ovaries of 14.5 dpc were cultured for 4 days. E_2_ and DPN significantly delayed prophase I progression. B. Ovaries of 14.5 dpc were cultured for 1 day. No significant differences were found among the groups. C. Ovaries of 17.5 dpc were cultured for 2 days. No significant differences were found between the group.

**
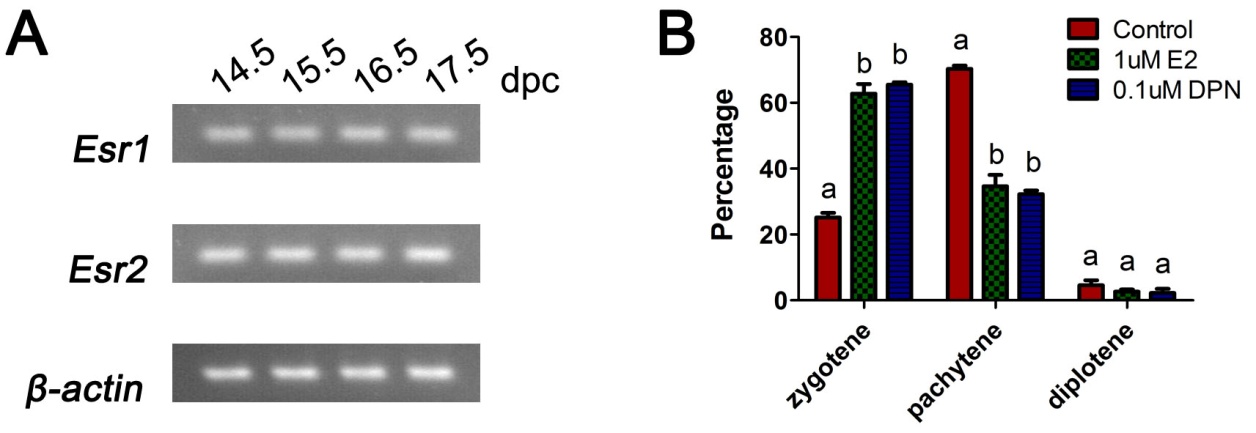
Supplementary figure 2.** **ERβ regulates meiotic prophase I of fetal oocytes.** A. The expressions of ERα and ERβ mRNA in the developing fetal ovary by gel electrophoresis. B. The comparison of the effect of E_2_ and DPN on the prophase I progression. Ovaries of 14.5 dpc were cultured for 3 days.


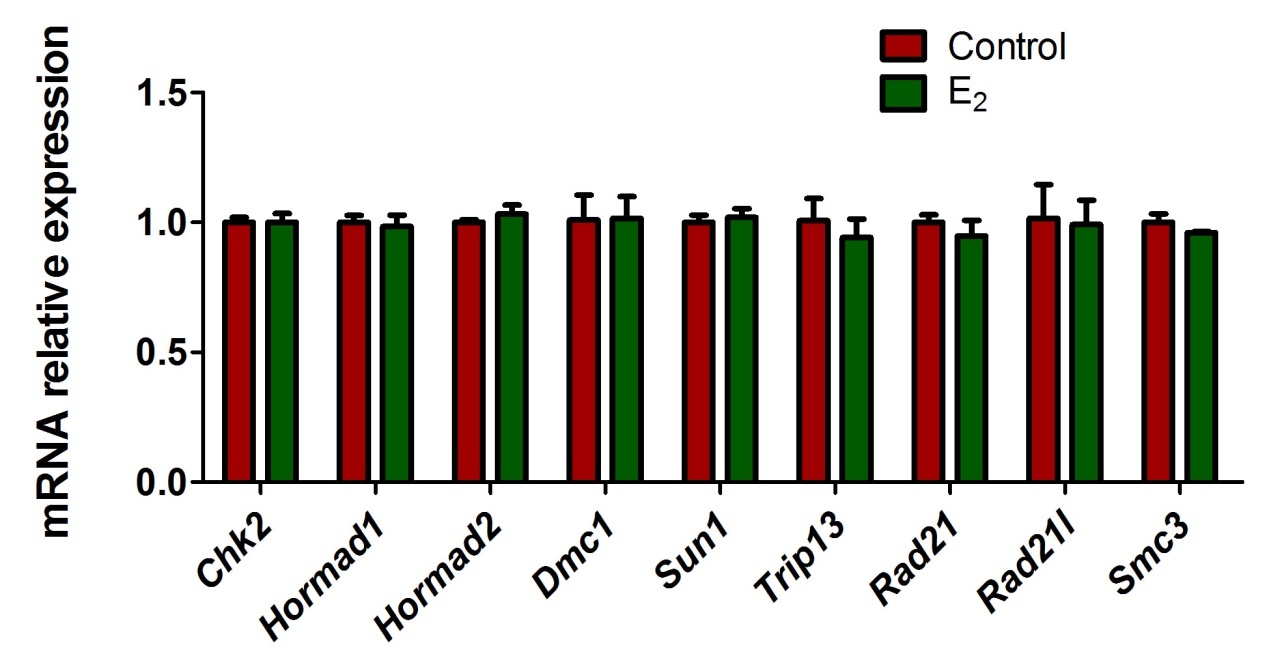
**Supplementary figure 3. The effect of E2 on meiotic key genes mRNA expression.** Ovaries of 14.5 dpc were cultured with or without E_2_ for 3 days.

**
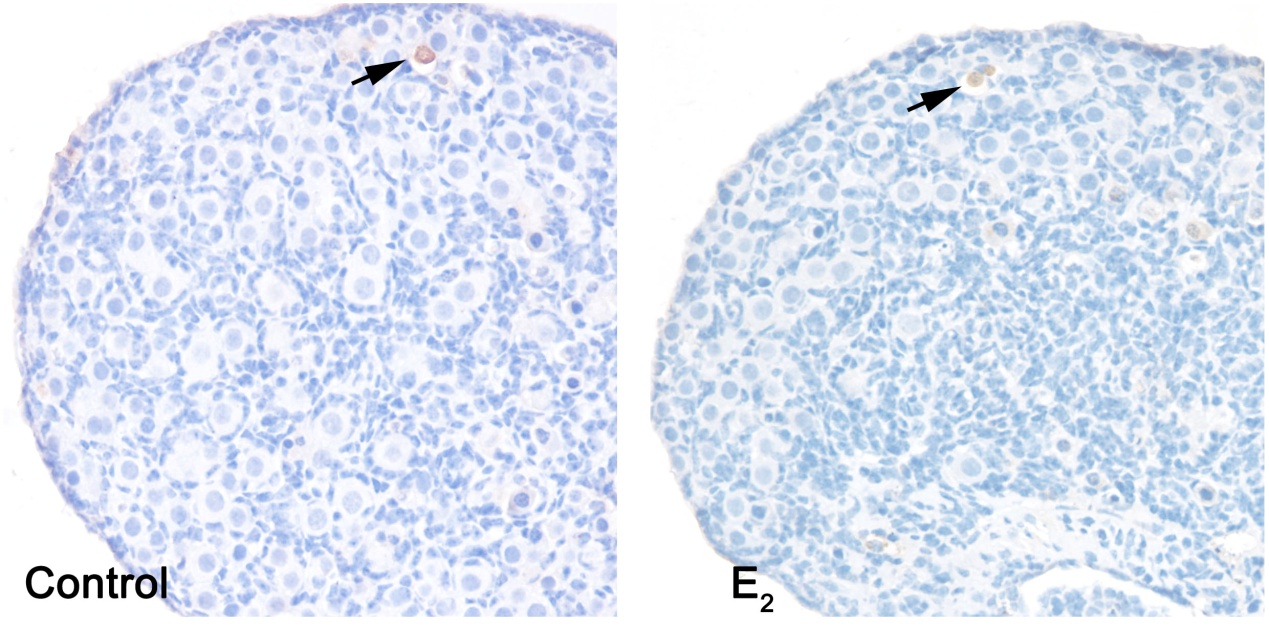
Supplementary figure 4. The apoptotic oocytes in the ovary cultures.** Ovaries of 14.5 dpc were cultured with or without E_2_ for 3 days, and cultured for another 5 days without the chemical. Then, the TUNEL assay was conducted. A. An section from a control ovary. B. An section from an ovary treated to E_2._ Arrows indicate apoptotic oocytes with DAB-stained nucleus.

**
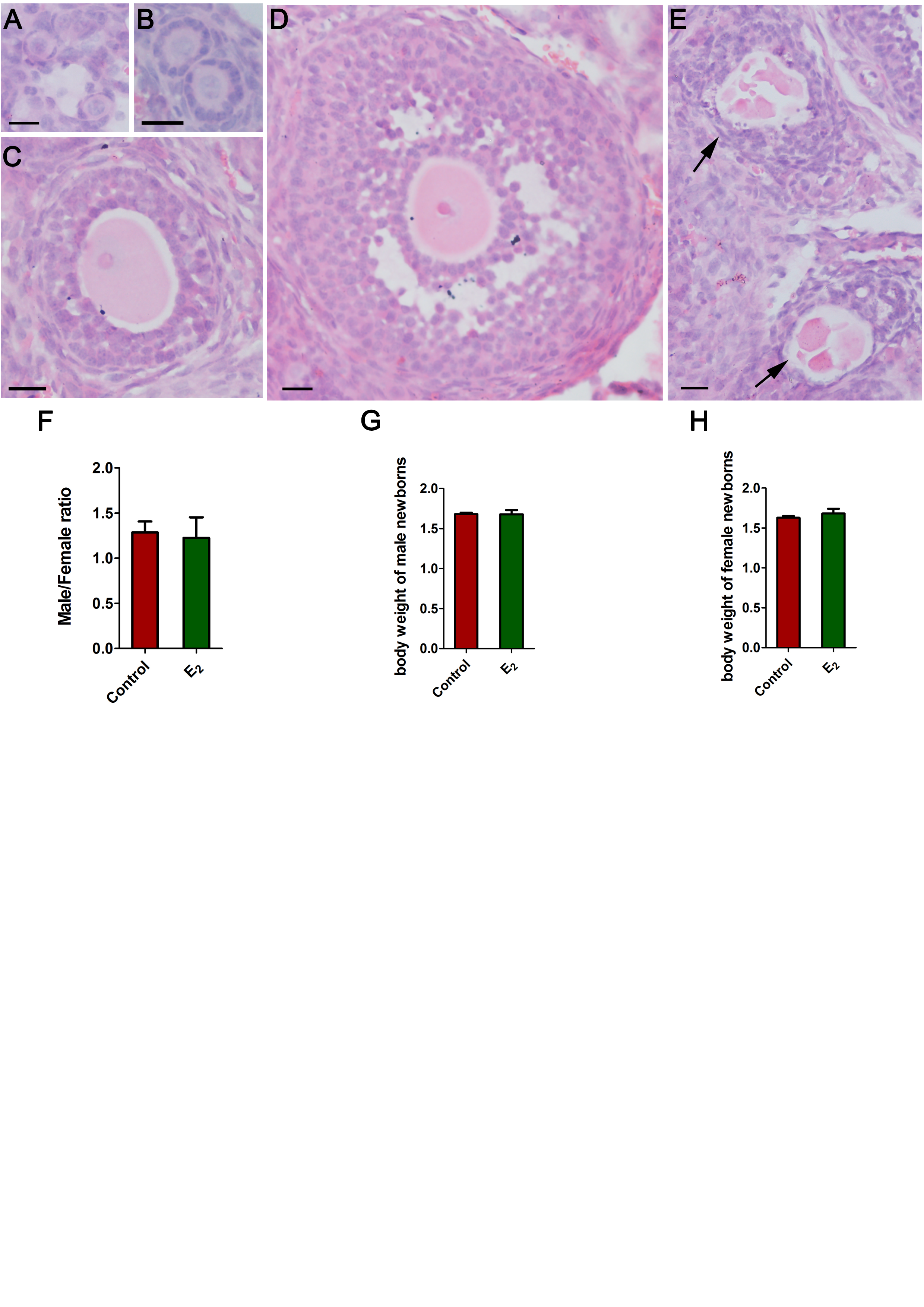
Supplementary figure 5. Assessment of long-term effect of maternal exposure to E_2_ on offspring fertility.** A-F: Prepubertal Female F1 offspring was injected with PMSG to stimulate follicular growth. Different follicle types in serial sections were analyzed. A. Primordial follicles: an oocyte surrounded by a single layer of flattened granulosa cells. B. Primary follicles: an oocyte surrounded by a single layer of cuboidal granulose cells. C. Secondary follicles: an enlarged oocyte surrounded by two more layers of granulosa cells. D. Antral follicle: follicles with antral spaces among granulose cells. E. Atretic follicle: fractured or shrinking oocytes were identified with abnormal follicle morphology. Arrows indicate fractured oocytes in atretic follicles. F-H: new born F2 pups. F. The male:female ratio. G. The birth weight of male pups. H. The birth weight of female pups. Bar: 10 μm.

**2 Supplementary Tables**

**Supplementary Table 1. Litter size of the F1 females.**


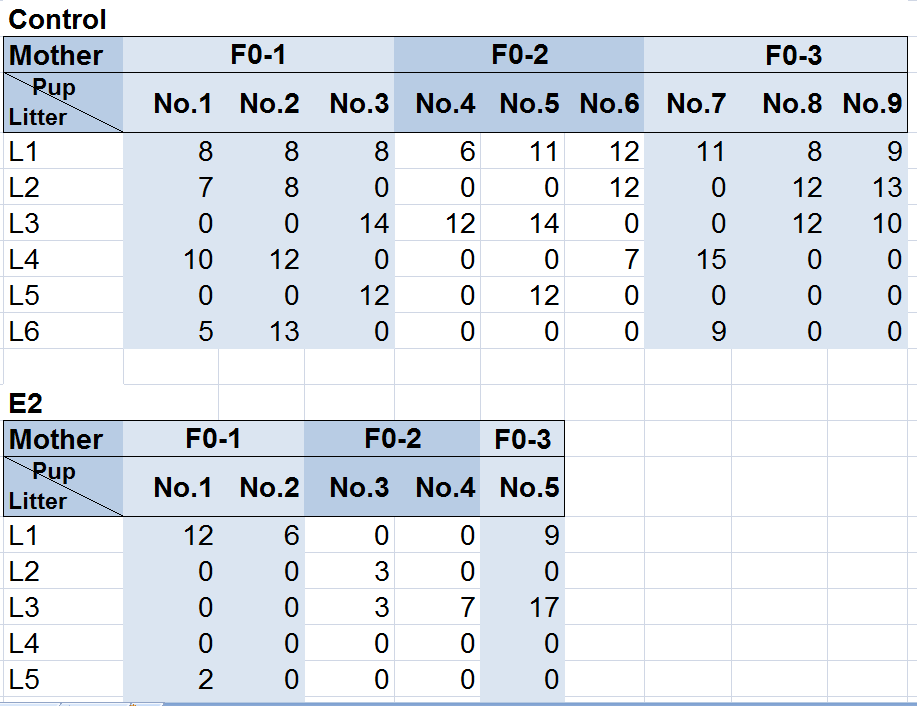


**Supplementary Table 2. The mixed model analysis for F1’s offspring.** The mixed model method was carried out by SPSS 16.0, with the total offspring number of F1 as the dependent variable, the treatments as the dependent variable, F0 mothers as the covariate. The covariance structure type adopts unstructured. It showed that the total number of offspring in the E_2_ group was significantly reduced than that of the control group.

|  | Estimate | SE | Df | t | p |
| --- | --- | --- | --- | --- | --- |
| E2 group VS. Control group | 25.17 | 8.209 | 11 | 3.066 | 0.011 |

**Supplementary Table 3. Assessment of follicle development in the ovaries of prepubertal F1 mice.**

**Supplementary Table 4. Primers**

| gene name | primer(5'-3') |
| --- | --- |
| *Atm* | sense: GATCTGCTCATTTGCTGCCG  antisense: GTGTGGTGGCTGATACATTTGAT |
| *Atr* | sense: GGTGTCACCAAAGAGGCGTAA antisense: GGGATTCGGCTTTCTGTTTCA |
| *Brca1* | sense: CTGCCGTCCAAATTCAAGAAGT  antisense: CTTGTGCTTCCCTGTAGGCT |
| *Dazl* | sense: GCAGCCACGTCCTTTGATTT antisense: TCATGGTTGGAGGCTGCAT |
| *Dmc1* | sense: CCTTTCAGGCTGATCCCAAA  antisense: CGTGAGCCAGAATGTGTCCA |
| *Polb* | sense: CATGCTCGTGGAACTCGCA  antisense: CAGATGCCGCTTTTCTGTACG |
| *Rec8* | sense: GAGCACCTACATCGTGCCC  antisense: CAGCCTCCTCCATATCAATGC |
| *Smc1b* | sense: GAAGATGGCATACGAGCCTTAG  antisense: CCATGCTCTATCCAAATCAGCC |
| *Smc3* | sense: TCCAGGGCTTCCGAAGTTAC  antisense: TTCTGCCCACGATGACATTATG |
| *Actb:* | sense: GGGAAATCGTGCGTGAC antisense: AGGCTGGAAAAGAGCCT |
|  |  |

**Supplementary Table 5**

| name | cat. No. | Company | Address | dilution |
| --- | --- | --- | --- | --- |
| anti-SYCP3 | sc-20845 | Santa Cruz | Dallas, USA | 1:50 |
| anti-SYCP3 | NB300-231 | Novus | Littleton, USA | 1:200 |
| anti-RAD51 | NB100-148 | Novus | CO, USA | 1:200 |
| anti-MLH1 | ab92312 | Abcam | San Francisco, USA | 1:200 |
| anti-γH2AX | JBW301 | Millipore | NewYork, USA | 1:400 |
| anti-ESR1 | MA-1-310 | Invitrogen | Shanghai, China | 1:30 |
| anti-ESR2 | NB200-305 | Novus | Littleton, USA | 1:200 |
| anti-DDX4 | ab27591 | Abcam | Shanghai, China | 1:200 |
| anti-POLβ | ABE1408 | Millipore | NewYork, USA | 1:1000 |
| anti-REC8 | ab19224 | Abcam | San Francisco, USA | 1:1000 |
| anti-TAF4b | obr313348 | Biorbyt | Wuhan, China | 1:500 |
| anti-FSHR | LS-C332397 | Lifespan biosciences | Seattle, USA | 1:500 |
| anti-cleaved caspase 3 | #9664 | Cell Signaling Technology | Danver, USA | 1:500 |
| Donkey anti -Rabbit IgG 488 | A-21206 | Life technologies | Rockford, USA | 1:200 |
| Donkey anti-goat IgG 555 | A-21432 | Life technologies | Rockford, USA | 1:200 |
| Donkey anti -mouse IgG 555 | A-31570 | Life technologies | Rockford, USA | 1:200 |
